# Comparative effectiveness of non-pharmacological interventions on depression and anxiety in aging populations: a systematic review and network meta-analysis of randomized controlled trials

**DOI:** 10.3389/fpsyt.2026.1772542

**Published:** 2026-06-10

**Authors:** Peifeng Qin, Fangbo Li, Xuecheng Li, Xiaowei Feng

**Affiliations:** 1 Guilin University of Technology at Nanning, Nanning, China; 2 South China Normal University, Guangzhou, China; 3 School of Football, Hainan Normal University, Hainan, China

**Keywords:** anxiety, dance therapy, depression, network meta-analysis, non-pharmacological interventions, older adults

## Abstract

**Background:**

Depression and anxiety are highly prevalent among older adults and significantly impair quality of life. Non-pharmacological interventions have increasingly gained attention as alternative or complementary therapeutic approaches. This study aimed to evaluate the comparative efficacy of these interventions for depression and anxiety in adults aged ≥55 years using a systematic review and network meta-analysis (NMA).

**Methods:**

Following PRISMA guidelines, five databases were systematically searched from inception to March 2025. Eighty-three randomized controlled trials (RCTs; n = 6,646) were included, covering 11 non-pharmacological interventions such as dance, qigong, cognitive behavioral therapy (CBT), internet-based CBT (ICBT), resistance training, and others. A random-effects NMA was conducted using Stata 15.1, with efficacy ranked using Surface Under the Cumulative Ranking Curve (SUCRA) values. Risk of bias was assessed using the Cochrane tool, and certainty of evidence was evaluated using the GRADE approach. Publication bias was examined using comparison-adjusted funnel plots and Egger’s regression tests (depression: intercept = 2.40, p = 0.029; anxiety: intercept = 1.00, p = 0.538).

**Results:**

Eighty-three RCTs (n = 6,646) encompassing 11 interventions were included. For depression, dance ranked highest (SUCRA = 98.5%) and demonstrated statistically significant superiority over CBT, ICBT, waitlist, and usual care. Resistance training (SUCRA = 67.5%) and CBT (SUCRA = 65.6%) ranked next. For anxiety, music (SUCRA = 78.9%) and Tai Chi (SUCRA = 77.1%) ranked highest; however, only Tai Chi and CBT showed statistically significant advantages over waitlist. MBSR demonstrated limited efficacy across both outcomes, while outdoor walking ranked among the least effective interventions for depression.

**Conclusions:**

Dance and music/Tai Chi demonstrated the highest probabilities of efficacy for depression and anxiety, respectively, followed by resistance training and CBT/ICBT. MBSR and outdoor walking showed limited effectiveness. Given the heterogeneity across studies, SUCRA rankings should be interpreted cautiously, and a personalized, stepped-care approach is recommended.

**Systematic review registration:**

https://www.crd.york.ac.uk/PROSPERO/view/, identifier CRD420251007063.

## 1 Introduction

Depression and anxiety are among the most prevalent mental health conditions affecting aging populations worldwide, with significant implications for individuals’ quality of life, physical health, and overall well-being (1, 2). According to global estimates, older adults are particularly vulnerable to these conditions, often due to factors such as social isolation (3), chronic illnesses, cognitive decline (4), and life transitions (e.g., retirement or bereavement) (5). Notably, even mild symptoms of depression and anxiety in this population can impair daily functioning and elevate the risk of more severe mental health disorders if left unmanaged (6). Therefore, addressing these challenges is a critical public health priority.

Pharmacological treatments, such as antidepressants and anxiolytics, are commonly prescribed for managing depression and anxiety (7, 8). However, these treatments are often associated with side effects, drug interactions, and limited long-term efficacy in older adults (9). Moreover, concerns about over-reliance on medication have prompted researchers and clinicians to explore non-pharmacological interventions as alternative or complementary approaches (10, 11). These interventions, which include psychosocial therapies, physical activity programs, mindfulness-based practices, and lifestyle modifications, aim to provide holistic and sustainable solutions for mental health management in aging populations (11, 12).

Non-pharmacological interventions encompass diverse modalities, such as music, dance, resistance training, meditation, and tai Chi (13, 14). The therapeutic mechanisms of these interventions vary, leading to differential efficacy in alleviating depressive and anxiety symptoms (15). While numerous randomized controlled trials (RCTs) have investigated the effectiveness of various non-pharmacological interventions, there remains a lack of consensus regarding their comparative effectiveness. Traditional pairwise meta-analyses often fail to account for the complexity and diversity of these interventions, leaving important questions unanswered about which strategies are most effective for specific subpopulations, such as those with or without mild mental health symptoms. Network meta-analysis (NMA), which allows the comparison of multiple interventions within a single analytical framework, offers a robust solution to this challenge by synthesizing both direct and indirect evidence (16).

This study presents a systematic review and network meta-analysis that aims to comprehensively evaluate the relative efficacy of non-pharmacological interventions in alleviating depressive and anxiety symptoms among older adults (≥55 years). The study population includes both healthy older adults and individuals presenting with mild mental health symptoms at baseline. By synthesizing direct and indirect evidence from 83 RCTs, this study seeks to provide a nuanced, evidence-based framework for guiding personalized mental health care strategies in older adults.

## 2 Methods

This review followed the guidelines of the PRISMA and this review registered the protocol with PROSPERO (CRD420251007063).

### 2.1 Search strategy

A systematic search of the existing literature on non-pharmacological interventions (resistance training, qigong, dance, music, tai chi, outdoor walking, mindfulness, and cognitive behavioral therapy) was completed on March 1, 2025. Five electronic databases including PubMed, Wiley online Library, Embase, Cochrane Library, and Web of Science (all collections) were systematically queried to ensure a thorough extraction of relevant studies. Three groups of search terms were used for the search: (1) (“aged”[MeSH] OR “aged, 80 and over”[MeSH] OR elderly OR “older adults”[tiab] OR geriatric*[tiab]); (2) (“internet-based cognitive behavioral therapy”[tiab] OR ICBT[tiab] OR “cognitive behavioral therapy”[tiab] OR CBT[tiab] OR “dance therapy”[MeSH] OR dance[tiab] OR “qigong”[MeSH] OR tai ji[tiab] OR taichi[tiab] OR “music therapy”[MeSH] OR music[tiab] OR “resistance training”[MeSH] OR “strength training”[tiab] OR “mindfulness-based stress reduction”[tiab] OR MBSR[tiab] OR “outdoor walk*”; (3) (“depressive disorder”[MeSH] OR depression[tiab] OR “anxiety disorders”[MeSH] OR anxiety[tiab]); and (4) (randomized controlled trial[pt] OR controlled clinical trial[pt] OR randomized[tiab] OR randomly[tiab]). Additionally, we reviewed the reference lists of all eligible articles, as well as reviews, for additional studies.

### 2.2 Inclusion and exclusion criteria

Following the population, intervention, comparison, and outcomes (PICO) framework, studies were eligible if they (1) studies involving older adults (≥ 55 years); healthy, or with mild mental health symptoms; (2) received non-pharmacological interventions (resistance training, qigong, dance, music, tai chi, outdoor walking, mindfulness, and cognitive-behavioral therapy) and had a comparison arm receiving either any intervention or usual care control or waitlist control; (3) the study must have evaluated one of the depression and anxiety symptoms; (4) the study reported sufficient data to compute pretest–posttest effect sizes. For published studies that did not provide the necessary statistics, the authors were contacted to obtain the relevant data; (5) We only included randomized controlled trials (RCTs).

Studies were excluded if they (1) were editorials, theses, poster or conference abstracts or presentations, or opinion pieces; (2) interventions involving any type of nutritional or pharmacological; (3) recruited older adults with Chronic or major diseases; (4) studies in which important information was lacking or the full text was not available.

### 2.3 Study selection

Upon reviewing the search outputs from the five databases, duplicates were eliminated prior to the examination of the results. Subsequently, the titles and abstracts, were evaluated against the predefined inclusion criteria to ascertain their relevance to the research question at hand. Following this, the complete texts of the articles that remained were scrutinized by two independent reviewers to determine their suitability for inclusion in the network meta-analysis. In cases of disagreement on study inclusion, the third author was consulted for consensus.

### 2.4 Data extraction process

Extracted data included the following information: first author, year of publication, participant characteristics, number of samples at end of treatment, interventions, duration, outcomes, and follow-up. Two reviewers independently extracted the aforementioned information. Disagreements were resolved through consultation with a third researcher.

### 2.5 Risk of bias assessment

The methodological quality of the included randomized controlled trials was assessed using the Cochrane Risk of Bias tool (RoB1.0), which evaluates seven domains: random sequence generation, allocation concealment, blinding of participants and personnel, blinding of outcome assessment, incomplete outcome data, selective reporting, and other sources of bias. All assessments were conducted independently by two reviewers, and any disagreements were resolved by a third reviewer.

The certainty of evidence for each primary comparison was evaluated using the GRADE (Grading of Recommendations, Assessment, Development and Evaluations) approach, which assesses five domains: risk of bias, inconsistency, indirectness, imprecision, and publication bias. The certainty of evidence was rated as high, moderate, low, or very low. All GRADE assessments were performed independently by two reviewers.

### 2.6 Statistical analyses

All statistical analyses were performed using Stata 15.1. A network meta-analysis (NMA) was conducted to compare the relative efficacy of different non-pharmacological interventions for depression and anxiety in older adults. Given that some studies reported pre- and post-intervention measures, we followed the methods outlined in the Cochrane Handbook to calculate the mean change and its standard deviation (SD) (17). Given the substantial clinical and methodological diversity of the included non-pharmacological interventions, a random-effects model was appropriately applied using the (network meta) command to estimate treatment effects. A network plot was generated to visualize the direct and indirect comparisons across interventions.

To assess the consistency of the network meta-analysis, an inconsistency test was conducted using the (network meta i) command.

The relative ranking of interventions was determined using Surface Under the Cumulative Ranking Curve (SUCRA) values. The (network rank) command was executed with 5000 repetitions to ensure robustness in ranking estimates. Higher SUCRA values indicated greater effectiveness. The probability distributions of rankings were visualized through probability distribution plots. A league table (net league) was generated to facilitate structured comparisons of effect sizes among interventions, with treatments ranked in descending order of effectiveness.

To assess small-study effects across the network, a comparison-adjusted funnel plot was generated, and a comparison-adjusted Egger regression test was applied (18). This test regresses the standardized effect estimate (Hedges’ g divided by its standard error) on study precision (1/SE), after centering each contrast on its network-estimated effect, and operates across all pairwise contrasts simultaneously (depression network: k = 76; anxiety network: k = 43). Unlike conventional per-comparison Egger tests, this approach is not constrained by the minimum 10-study threshold typically applied to individual comparisons. A global trim-and-fill method was not applied because it assumes a single symmetric funnel and lacks a validated extension for multi-treatment network meta-analysis (19, 20).

## 3 Results

**Figure 1:**
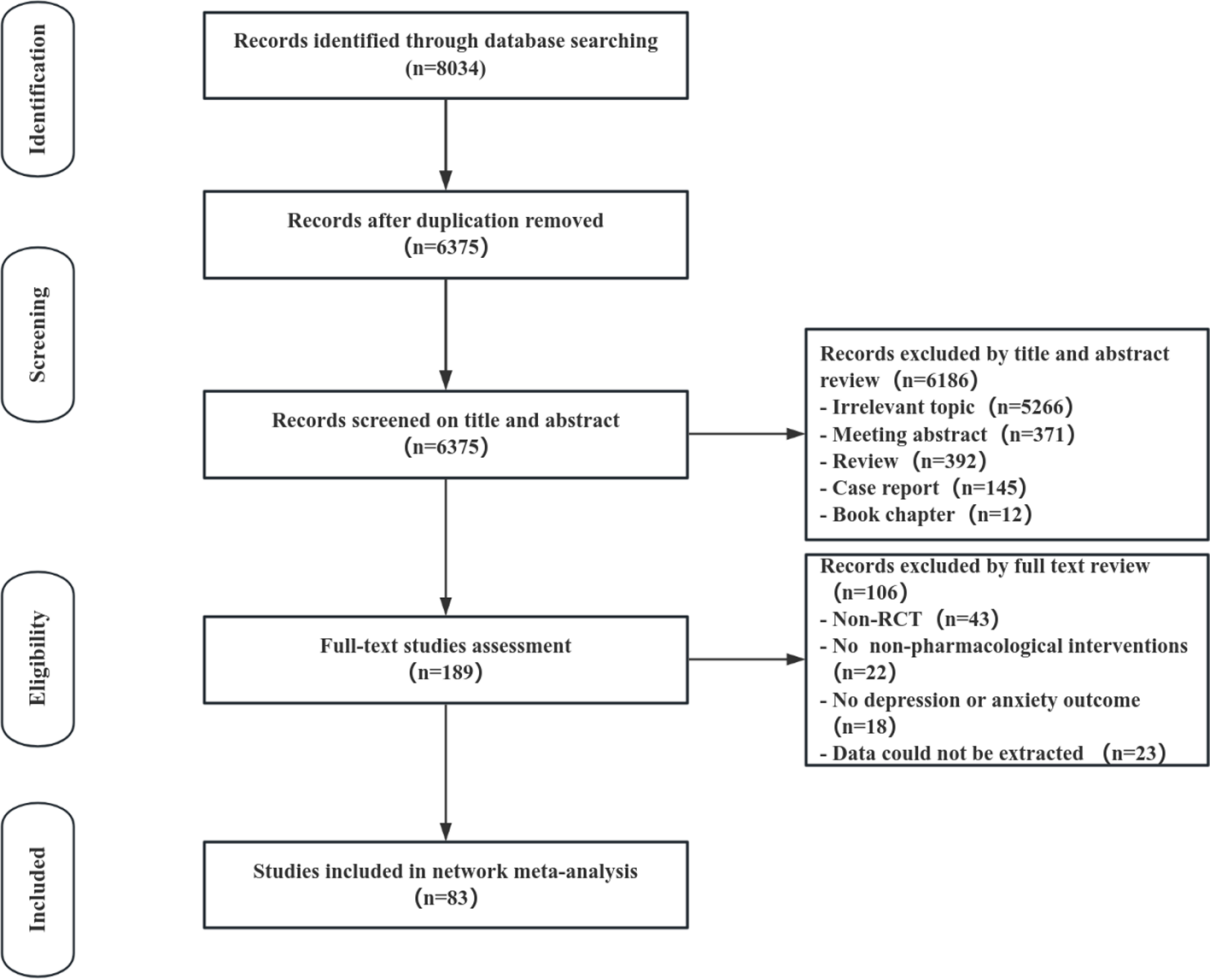
PRISMA flow diagram of study selection.

Overall, 8,034 records were initially identified from electronic databases. After removing 1,659 duplicates, we filtered 6375 records based on reading titles and abstracts. Finally, 83 randomized controlled trials met the eligibility criteria and were included (**Figure 1**).

### 3.1 Study characteristics

**Figure 2:**
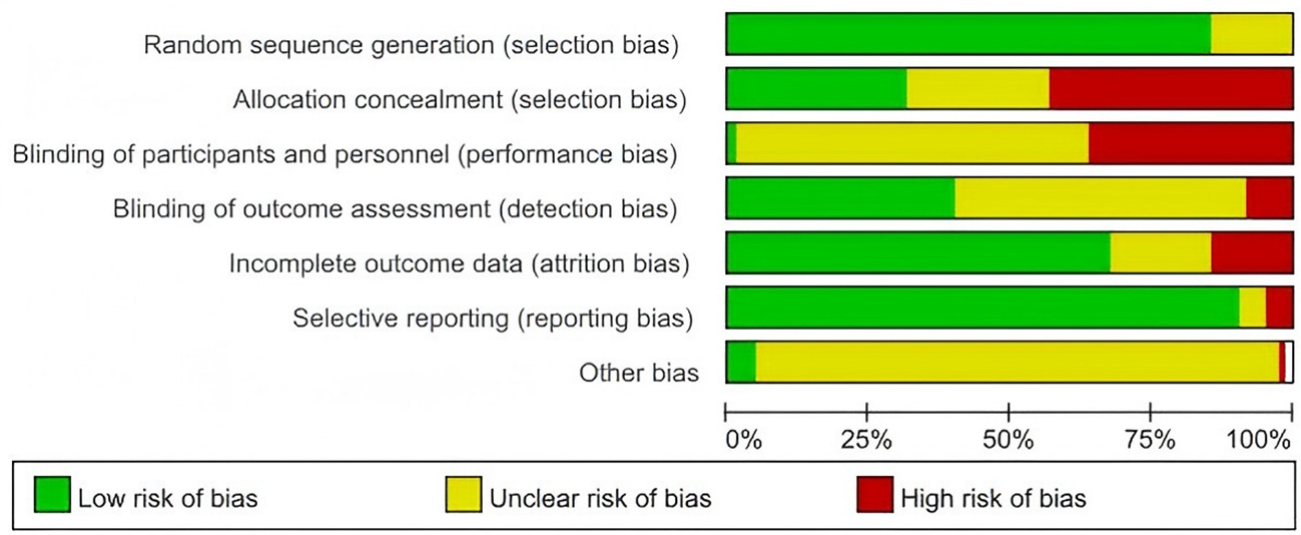
Risk of bias assessment of included studies.

The 83 studies from 21 regions included in the systematic review involved 6646 participants. The RCTs’ sample sizes ranged from 8 to 315 participants. Interventions included resistance training (21–37), qigong (38–43), dance (44–47), music (48–53), tai chi (54–65), outdoor walking (66–68), mindfulness (69–80), CBT (39, 57, 69, 81–93) and ICBT (83, 94–101). The mean duration of interventions was 11.1weeks. 64/83 studies (about 77%) reported the follow-up time (**Table 1**). **Figure 2** presents the risk-of-bias assessment of the included studies. While random sequence generation was adequately reported in the majority of studies (86% low risk), allocation concealment emerged as the most prominent methodological concern, with 43% of studies rated as high risk. Blinding of participants and personnel was rated as high risk in 36% of studies, which is largely inherent to the nature of physical and behavioral interventions. Blinding of outcome assessors was rated as high risk in 8% of studies and unclear in 52%. Incomplete outcome data and selective reporting were generally well addressed, with low risk observed in 68% and 91% of studies, respectively. Overall, the most substantial methodological concerns were related to allocation concealment and blinding of participants.

**Table 1:** Baseline characteristics of included studies.

No.	Author, years	Country	Population characters	Number analyzed at end of treatment (N)	Intervention	Duration	Main outcomes	Follow-up
1	Almeida, 2021 (102)	Australia	adults aged 65 years	153 usual care and 154 BA	behavioral activation (BA) program	8 weeks	GAD-7、PHQ-9	None specified
2	Ansai, 2015 (21)	Brazil	Sedentary older adults aged 80 years and older, community-dwelling individuals	(control, multicomponent training or resistance training) (n = 23 per group)	Multicomponent training vs. resistance training vs. control group	16 weeks	GDS	None specified
3	Baklouti, 2023 (103)	Tunisia	Individuals above the age of 65, of both genders	160 (Yoga: 65, Control: 95)	Yoga vs waitlist	8 weeks	DASS-21	None specified
4	Biasutti, 2019 (48)	Italy	Elderly people (aged 62–95) with and without cognitive impairment, living in care homes	45 (20 in experimental group, 25 in control group)	Music training consisting of improvisational exercises and rhythm-melodic exercises	6 weeks	GDS	None specified
5	Cassilhas, 2010 (23)	Brazil	Sedentary elderly men aged 65 to 75 years	43 participants (23 in control group, 20 in exercise group)	High resistance exercise vs. control group	24 weeks	STAI-S	None specified
6	Chan, 2017 (38)	China	Hidden elderly (aged ≥ 60 years, socially isolated, no social activity participation)	46 (Qigong: 24, Control: 22)	Qigong	12 weeks	MhI-18	3 months, 6 months
7	Chen, 2016 (82)	China	Chinese older adults with generalized anxiety disorder (GAD), aged 60 years or older	32 (CBT-IU group), 31 (Control group)	Group Cognitive Behavioral Therapy	12 weeks	BAI、BDI	6 months
8	Chen, 2016 (49)	UK	Elderly people (aged 60+) from general population	258 (131 in experimental group, 121 in control group)	Community singing groups led by professional musicians (compared to usual activities)	14 weeks	HADS	6 months
9	Cunha, 2022 (24)	Brazil	Trained older women aged 60 years and older, physically independent	Four different resistance exercise (n = 15 per group)	resistance exercise	12 weeks	BAI、GDS	12 weeks post-intervention
10	Damush, 1999 (25)	USA	Community-dwelling older adult women, mean age 68 years	62 participants (33 in exercise group, 29 in control group)	Resistance training using elastic bands vs. control group	8 weeks	HRQOL	None specified
11	Danieli, 2022 (94)	Italy	Aging adults with stress and anxiety (mean age = 55.58 years, 78% female, 55+ years old)	4 groups (SMT-CBT:16, SMT-CBT-PHA:16, PHA-onlt:14, Test-only:14)	Mobile personal health care agent (TEO) with conversational AI	8 weeks	SCL-90-R	3 months
12	Danny, 2022 (68)	China	older adults with depression (aged ≥ 50 years, ethnic Chinese, diagnosed with clinical depression)	30 (CON: 10, MOD: 10, VIG: 10)	Moderate walking exercise	12 weeks	BDI、GAD-7	None specified
13	Titov, 2015 (101)	Australia	Older adults with symptoms of depression (mean age = 65.31 years, 82% female, 60+ years old)	treatment group (n = 29);waitlist control group (n = 25)	Internet-delivered cognitive behavior therapy (ICBT)	8 weeks	GAD-7、PHQ-9	3 months and 12 months
14	Dear, 2015 (95)	Australia	Older adults with symptoms of anxiety (mean age = 65.39 years, 67% female, 60+ years old)	treatment group (n = 35);waitlist control group (n = 37)	ICBT	8 weeks	GAD-7、PHQ-9	3 months and 12 months
15	DiNapoli, 2017 (81)	USA	Rural, ethnically diverse older adults with elevated psychological symptoms and decreased quality of life, aged 65 years or older	70 (CBT group), 64 (Minimal Support Control group)	Home-delivered Cognitive Behavioral Therapy (CBT)	13 weeks	SCL-90-R	None specified
16	Eyigor, 2009 (44)	Turkey	Healthy adult elderly females over the age of 65 years, living independently	37 (Group 1: 19, Group 2: 18)	Turkish folkloric dance exercise program vs. no exercise (control).	8 weeks	GDS	8-week post-intervention assessment
17	Fraile, 2024 (22)	Spain	Older adults aged 65 or older, community-dwelling	116 participants (57 in experimental group, 59 in control group)	Combined resistance training and Mediterranean diet vs. control group	12 weeks	HADS	None specified
18	Galinha, 2021 (50)	Portugal	Elderly adults (aged 60–95) from a social care institution	89 in intervention group, 60 in wait-list control group at baseline (ITT analysis)	4-month singing program	16 weeks	DASS-21	6 months
19	Ge, 2021 (54)	China	Prefrail elderly in senior living communities, aged 60-89	TCG: 32, CG: 33	Short 8-form Tai Chi vs. usual care	8 weeks	GDS	4 weeks, 8 weeks
20	He, 2024 (44)	Hong Kong	Older adults with sleep disturbance, aged ≥ge	TC+active rTMS: 38, TC+sham rTMS: 38, TC: 38, PE: 38	Tai Chi + rTMS vs. Tai Chi alone vs. PE	4 weeks	DASS-21	Post-intervention, 3-month follow-up
21	Helen, 2023 (58)	USA	Depressed older adults, aged ≥ge	TCC: 62, HEW: 63	Tai Chi Chih (TCC) vs. Health Education and Wellness (HEW)	12 weeks	GDS	3 months, 6 months
22	Hola, 2024 (45)	Czech Republic	Independently living older adults, aged 65–80 years, non-obese, higher education levels	77 (Dance group: 20, Martial arts group: 16, Control group: 23)	Dance vs. Martial arts vs. Control (waiting list)	12 weeks	GDS	12-week post-intervention assessment
23	Hsu, 2016 (56)	China	Older people using wheelchairs in long-term care	Tai Chi: 30, Control: 30	Seated Tai Chi vs. usual activities	26 weeks	GDS	Midpoint (13 weeks), End of intervention (26 weeks)
24	Irwin, 2014 (57)	USA	Older adults with chronic and primary insomnia, aged ≥ge	CBT: 50, TCC: 48, SS: 25	CBT vs. Tai Chi Chih vs. Sleep Seminar (SS)	16 weeks	IDS-C	7 months, 16 months
25	Jansen, 2016 (70)	Germany	55 adults aged 52–81 years, with subjective well-being, health, cognitive functioning, and chronic stress measured	55 participants (Karate: n=23, MBSR: n=23, Control: n=9)	Mindfulness-Based Stress Reduction (MBSR) No training (Control group)	8 weeks	HADS	None specified
26	Jing, 2018 (39)	China	Elderly housebound (aged ≥ 60 years, housebound for at least 6 months, no prior Baduanjin or CBT training)	79 (EG: 39, CG: 40)	Qigong	24 weeks	GDS	None specified
27	Jones, 2016 (96)	Canada	46 older adults (aged 60 years or older) with generalized anxiety disorder (GAD) or subclinical GAD	ICBT group: 22; Waiting list control (WLC) group: 19	ICBT vs. waiting list control	10 weeks	GDS	1-month follow-up
28	Kekäläinen, 2017 (26)	Finland	Men and women aged 65–75 years, community-dwelling	106 participants (26 in RT1, 27 in RT2, 28 in RT3, 25 in control group)	Resistance training	24 weeks	BDI	9 months
29	Kim, 2019 (27)	South Korea	Elderly women aged 67 to 81 years, sedentary	21 participants (11 in strength exercise group, 10 in control group)	Strength training program	24 weeks	SGDS-K	None specified
30	Kong, 2024 (83)	China	354 older adults (aged 60 years or older) with subthreshold depression (sD) in institutional long-term care settings	ICBT group: 105; Group-based CBT group: 104; Waiting list (WL) group: 106	ICBT vs. CBT vs. waiting list.	5 weeks	GAD-7、GDS	6-month and 12-month follow-ups
31	Lee, 2021 (72)	China	aged ≥ge years old	MBSR(n = 120) Waitlist control group(n = 123)	MBSR Waitlist control group	8 weeks	GDS	2 months after intervention for mMBSR group
32	Liao, 2018 (59)	China	Community-dwelling older persons with mild to moderate depressive symptoms	Music+Tai Chi: 55, Control: 52	Combined music and Tai Chi vs. routine health education	12 weeks	GDS	Monthly for 3 months
33	Liu, 2016 (60)	China	Healthy elderly women aged 60–70 years, no significant chronic diseases, sedentary lifestyle.	63 (Tai Chi group = 32, Control group = 31)	24-Form Simplified Tai Chi exercise	16 weeks	SCL-90-R	8 weeks post-intervention
34	Liu, 2018 (61)	China	Elderly individuals with depression (GDS score ≥core aged ≥ge years, sedentary lifestyle.	60 (Tai Chi group = 30, Control group = 30)	24-Form and 42-Form Tai Chi	24 weeks	GDS	None specified
35	Mallya, 2016 (74)	Canada	97 healthy older adults (age ≥ag years)	97 participants (MBSR: n=57, Active control: n=40)	MBSR Active control group	8 weeks	GDS	None specified
36	Mallya, 2016 (75)	UK	147 participants with subjective cognitive decline (SCD), mean age 72.7 years	147 participants (CMBAS: n=73, HSMP: n=74)	Caring Mindfulness-Based Approach for Seniors Health Self-Management Program	8 weeks	STAI-S、GDS	6 months
37	Marianne, 2014 (67)	Canada	Postmenopausal women (aged 52–69 years, BMI 22–29 kg/m², sedentary, no hormone therapy)	23 (OUT: 12, IN: 11)	Outdoor walking	12 weeks	BDI	None specified
38	Martins, 2011 (28)	Portugal	Older adults (65–95 years old), living in a care home, sedentary lifestyle, no severe diseases	78 (Control group = 31, Aerobic Training (AT) group = 24, Strength Training (ST) group = 23)	Aerobic training (AT) and strength training (ST) compared to a control group	16 weeks	POMS-SF	None specified
39	MD, 2014 (46)	Czech Republic	Older adults (60 years or older) permanently living in nursing homes, with various levels of cognitive function (MMSE ≥ 15)	162 (Intervention group: 79, Control group: 83)	Dance therapy (Exercise Dance for Seniors - EXDASE) vs. regular activities (control)	12 weeks	GDS	3-month post-intervention assessment
40	MD, 2014 (85)	USA	Anxious older adults with generalized anxiety disorder (GAD), aged 60 years or older	30 (Intact EF group:10, Improved EF group:10, ExecDys group:10)	CBT	13 weeks	BAI、BDI	12 months
41	Mohlman, 2008 (84)	USA	Older adults with generalized anxiety disorder (GAD) and low scores on executive skills tests, aged 60 years or older	4 (CBT group), 4 (CBT/APT group)	Standard CBT	16 weeks	BAI、BDI	6 months
42	(Moraes, 2019) (29)	Brazil	Older adults with major depressive disorder (iso years old), receiving antidepressant treatment, no severe cognitive impairment or other severe diseases	27 (Aerobic Training (AT) group = 9, Strength Training (ST) group = 9, Control group = 9)	Aerobic training (AT) and strength training (ST) compared to a low-intensity exercise control group	12 weeks	BDI	None specified
43	Morton, 2016 (97)	UK	Older adults receiving care (mean age = 80.71 years, 50% female, 60–95 years old)	(training: 53, control: 44)	Internet connectivity and training for social purposes	12 weeks	CES-D、GAI	4 months
44	Moss, 2014 (76)	USA	39 elderly residents in a continuing care retirement community, mean age 82 years	39 participants (MBSR: n=20, Waitlist control: n=19)	Adapted MBSR Waitlist control group	8 weeks	BSI-18	None specified
45	Noradechanunt, 2016 (62)	Australia	Healthy but sedentary adults aged >60 years	39 (Thai Yoga = 13, Tai Chi = 13, Control = 13)	Thai Yoga and Tai Chi	12 weeks	CES-D	12 weeks post-intervention
46	Oh, 2017 (98)	South Korea	Older adults with subjective memory complaints (mean age = 59.30 years, 70% female, 50–68 years old)	SMART (n: 18) vs. Fit Brains (n: 19) or a wait-list group (n: 16)	Smartphone-based memory training (SMART vs. Fit Brains™)	8 weeks	STAI-S、CES-D	None specified
47	Peixoto, 2024 (30)	Brazil	Women, ≥om years old, normal cognition, sedentary lifestyle	29 (Experimental group = 15, Control group = 14)	Experimental group performed combined cognitive and physical exercises, control group performed stretching exercises	10 weeks	GDS	None specified
48	Romero, 2019 (77)	Spain	Community-dwelling older people aged >65 years with loneliness identified by primary care team	55 (29 in intervention group, 26 in control group)	Mindfulness	24 weeks	YAQ	None specified
49	Ruth, 2018 (66)	Canada	Older adults (aged ≥ 65 years) who infrequently walk outdoors (u 20 min/week)	9 (GO-OUT: 6, Workshop: 3)	GO-OUT program	12 weeks	GDS	6 months
50	Sahin, 2018 (31)	Turkey	Institutionalized frail elderly (lde years old), meeting Fried criteria for frailty, no severe diseases	48 (High Intensity (HI) group = 16, Low Intensity (LI) group = 16, Control group = 16)	High-intensity (70% 1RM) and low-intensity (40% 1RM) strength training	8 weeks	GDS	None specified
51	Lwi, 2022 (73)	USA	Healthy older adults aged 60–80 years	49 (23 in MBSR group, 26 in Brain Health group)	MBSR class vs. Brain Health education class	8 weeks	STAI-S、GDS	3 months
52	Seeley, 2016 (86)	USA	Older adults with mild to moderate depression and/or anxiety, aged 55 years or older	31 (peer-facilitated CBT group), 31 (wait-list control group)	CBT	10 weeks	GAD-7、PHQ-9	None specified
53	Shafran, 2022 (69)	Israel	Healthy older adults aged ≥ge years	22 (7 in MBIS group, 8 in CBT group, 7 in control group)	MBIS vs. CBT for anxiety vs. care-as-usual control	8 weeks	BAI、PHQ-9	None specified
54	Shapira, 2021 (78)	Israel	Community-dwelling older adults aged 65–90 years	64 (intervention group), 9 (control group)	CBT and mindfulness skills	3.5 weeks	PHQ-9	1-month follow-up
55	Shih, 2021 (79)	China	Older adults aged ≥ge years with mild to moderate depressive symptoms	24 (MBCT group), 25 (active control group)	MBCT vs. active control (physical exercise and health education)	8 weeks	HDRS	None specified
56	Silfvernagel, 2018 (99)	Sweden	66 older adults (aged over 60 years) with mixed anxiety and depression	Treatment group: 22; Control group: 33	ICBT vs. weekly general support	8 weeks	GAD-7、MADRS	1-year follow-up
57	Sims, 2006 (32)	Australia	≥us years old, with depressive symptoms, not receiving antidepressant treatment	32 (Strength Training group = 14, Control group = 18)	Community-based strength training program	10 weeks	GDS	6 months
58	Singh, 1997a (33)	USA	≥SA years old, with mild or major depressive symptoms, not receiving antidepressant treatment	32 (Strength Training group = 17, Control group = 15)	High-intensity strength training	10 weeks	GDS	None specified
59	Singh, 1997b (34)	USA	≥SA years old, with mild or major depressive symptoms, not receiving antidepressant treatment	28 (Strength Training group = 15, Control group = 13)	High-intensity strength training	10 weeks	GDS	None specified
60	Singh, 2005 (35)	Australia	≥us years old, with mild or major depressive symptoms, not receiving antidepressant treatment	54 (High Intensity group = 20, Low Intensity group = 20, Control group = 14)	High-intensity (80% 1RM) and low-intensity (20% 1RM) strength training	8 weeks	GDS	None specified
61	Smith, 2021 (87)	Australia	Community-dwelling older adults with comorbid depression and anxiety, aged 60 years or older	27 (CBT group), 35 (waitlist control group)	CBT	12 weeks	GAI、GDS	3 months
62	Solianik, 2021 (63)	Lithuania	Elderly adults aged 60–78 years, sedentary lifestyle, no significant chronic diseases	30 (Tai Chi group = 15, Control group = 15)	8-Form Tai Chi	10 weeks	PSS-10	None specified
63	Song, 2022 (64)	China	Elderly women with knee osteoarthritis (KOA), aged 60–75 years	35 (Tai Chi group = 18, Control group = 17)	Modified Tai Chi	12 weeks	SAS、SDS	3 months and 6 months
64	Stanley, 1996 (88)	USA	Adults aged 55+, diagnosed with GAD	31 (CBT: 18, SP: 13)	CBT	14 weeks	STAI-TRAIT、BDI	6 months
65	Stanley, 2003 (89)	USA	older adults (age 60 years and over) with generalized anxiety disorder	CBT (n = 39, MCC (n = 41)	CBT	14 weeks	STAI-TRAIT、BDI	6 months
66	Stanley, 2009 (91)	USA	Adults aged 60+, diagnosed with Generalized Anxiety Disorder (GAD)	CBT (n = 70, EUC (n = 64)	CBT	12weeks	GADSS、BDI	12 months
67	Stanley, 2014 (90)	USA	older adults (mean age, 66.9 years) with GAD recruited from primary care clinics at two sites	BLP (n = 76), PLP (n = 74), or UC (n = 73)	CBT	12weeks	STAI-TRAIT、PHQ-8	None specified
68	Stenlund, 2009 (40)	Sweden	Patients with burnout (aged 65 years, diagnosed with exhaustion syndrome)	68 (Qigong: 33, Control: 35)	Qigong	12 weeks	HADS	None specified
69	Mathew, 2017 (52)	India	Institutionalized geriatric adults (aged 65+) with mild depression	40 in intervention group, 40 in control group	Daily group singing sessions led by a music therapist	3 weeks	GDS	None specified
70	Thorén, 2014 (100)	Sweden	Experienced hearing-aid users (mean age = 69.3 years, 47% female, 26–81 years old)	Intervention group (n = 38);Control group (n = 38)	Online rehabilitative intervention	5 weeks	HADS	3 months
71	Tsang, 2003 (42)	China	Elderly with chronic physical illnesses (aged ≥ 65 years, sub-acute stage of chronic illness)	50 (Qigong: 24, Control: 26)	Qigong	12 weeks	GDS	None specified
72	Tsang, 2006 (41)	China	Elderly with depression (aged ≥ 65 years, diagnosed with depression or depressive features)	82 (Qigong: 48, Comparison: 34)	Qigong	16 weeks	GDS	4 weeks,8weeks
73	Tsang, 2006 (43)	China	Depressed elders with chronic illness (aged ≥ 65 years, diagnosed with major depression disorder)	36 (Qigong: 21, Comparison: 17)	Qigong	12 weeks	GDS	4 weeks,8weeks
74	Tsang, 2006 (46)	Japan	≥ap years old, sedentary lifestyle, healthy but inactive older women	36 (High Intensity group = 12, Moderate Intensity group = 12, Control group = 12)	High-intensity (75-85% 1RM) and moderate-intensity (55-65% 1RM) strength training	12 weeks	MS	None specified
75	Ugur, 2017 (51)	Turkey	Elderly people (mean age 75–78) living in a nursing home	32 in music group, 32 in control group	Listening to Turkish traditional music and Sufi music	8 weeks	GDS	None specified
76	Wetherell, 2003 (92)	USA	Older adults with generalized anxiety disorder	CBT (n: 26) or DG (n: 26) or a 12-week waiting period (n: 23).	CBT	12 weeks	BDI、BAI	6 months
77	Witlox, 2021 (93)	Netherlands	Adults aged between 55–75 years with mild to moderately severe anxiety symptoms	CBT (n = 164, ACT (n = 150)	CBT	12 weeks	GAD-7、PHQ-9	6 months,12 months
78	Yang, 2019 (53)	China	Elderly patients (aged 60–88) with depression	47 in treatment group, 23 in Western music group, 21 in control group	Listening to traditional Chinese five-elements music	4 weeks	SCL-90-R、SDS	None specified
79	Yıldırım, 2016 (65)	Turkey	Healthy elderly adults aged 55–76 years, sedentary lifestyle	48 (Tai Chi group = 27, Combined exercise group = 21)	Tai Chi vs. Combined exercise prescription (walking, strength training, flexibility)	12 weeks	GDS	None specified
80	YILDIZ, 2023 (71)	Turkey	Older adults aged ≥ge years living in a nursing home	24 (intervention group), 24 (control group)	MBSR program	8 weeks	GDS	1-month follow-up
81	Zanuso, 2012 (37)	UK	≥K1 years old, healthy older adults, no exercise program in the past 5 years	20 (Exercise group = 10, Control group = 10)	Moderate-intensity (80% of 5RM) strength training	12 weeks	POMS-SF、TAI	None specified
82	Zhang, 2015 (80)	China	Adults aged ≥ge years with chronic insomnia	29 (MBSR group), 28 (wait-list control group)	MBSR program	8 weeks	GDS、SAS	None specified
83	Zhang, 2015 (47)	China	Chinese older adults living in the community, aged 60 years or above, with frailty index ≥ 0.23	72 (Dance group: 36, Control group: 36)	Square dance intervention vs. normal daily activities (control)	16 weeks	GDS	16-week post-intervention assessment

GAD-7, Generalized Anxiety Disorder 7-item scale; PHQ-9, Patient Health Questionnaire-9; DASS-21, Depression Anxiety Stress Scales-21 items; GDS, Geriatric Depression Scale; STAI-S, State-Trait Anxiety Inventory - State subscale; BAI, Beck Anxiety Inventory; BDI, Beck Depression Inventory; HADS, Hospital Anxiety and Depression Scale; HRQOL, Health-Related Quality of Life; SCL-90-R, Symptom Checklist-90-Revised; CES-D, Center for Epidemiologic Studies Depression Scale; GAI, Geriatric Anxiety Inventory; IDS-C, Inventory of Depressive Symptomatology - Clinician-rated; BSI-18, Brief Symptom Inventory-18; POMS-SF, Profile of Mood States-Short Form; MADRS, Montgomery-Asberg Depression Rating Scale; PSS-10, Perceived Stress Scale-10 items; SAS, Self-Rating Anxiety Scale; SDS, Self-Rating Depression Scale; GADSS, Generalized Anxiety Disorder Severity Scale; PHQ-8, Patient Health Questionnaire-8; HDRS, Hamilton Depression Rating Scale; STAI-TRAIT, State-Trait Anxiety Inventory - Trait subscale; SGDS-K, Short Geriatric Depression Scale-Korean version; TAI, Trait Anxiety Inventory; MhI-18, Mental Health Inventory-18; YAQ, Yale Anxiety Questionnaire; MS, Mood Scale; CBT, Cognitive Behavioral Therapy; BA, Behavioral Activation; rTMS, repetitive Transcranial Magnetic Stimulation; TCC, Tai Chi; MBSR, Mindfulness-Based Stress Reduction; ICBT, Internet-delivered Cognitive Behavioral Therapy; ACT, Acceptance and Commitment Therapy; PE, Physical Exercise.

### 3.2 Network plot

**Figure 3:**
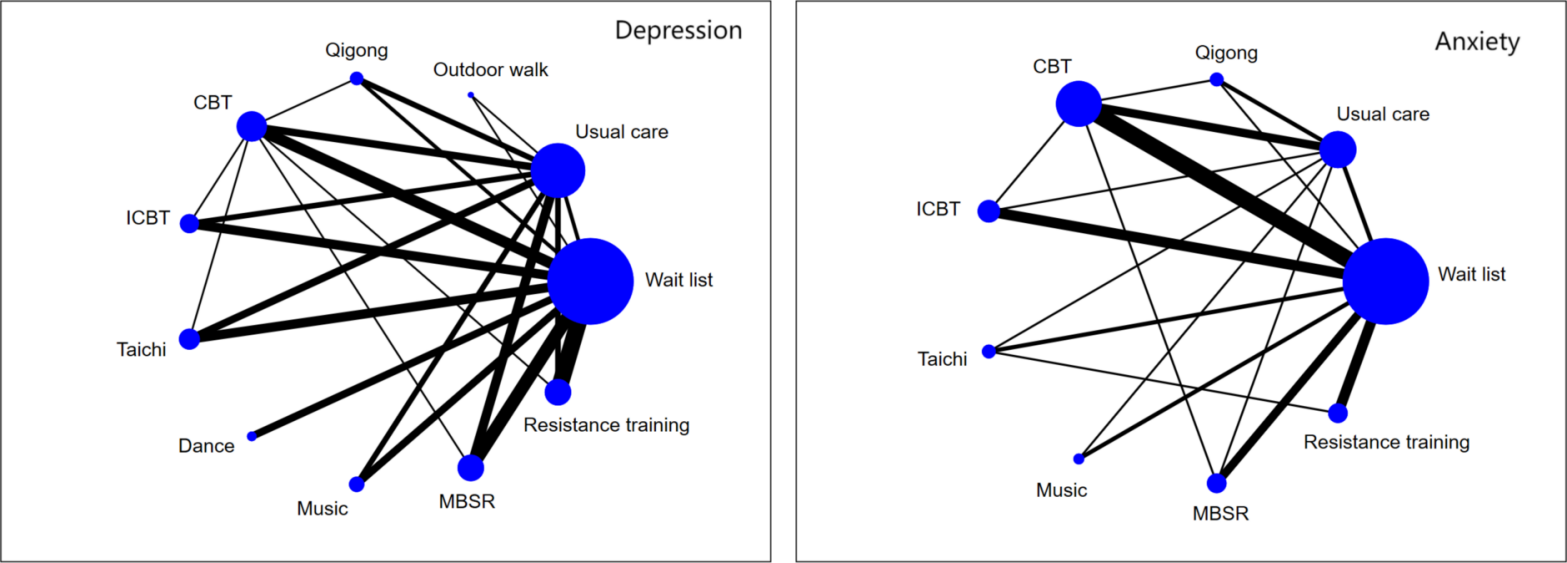
Network plots of interventions for depression and anxiety.

**Figure 3** presents the network plot of treatment comparisons for depression and anxiety. The network meta-analysis (NMA) for depression was conducted using data from all 83 included RCTs reporting depression outcomes. This yielded 87 study entries (accounting for multi-arm trial designs) with a total sample size of 5,596 participants. The analysis included 11 interventions, comprising 32 pairwise comparisons, of which 23 were direct comparisons. Similarly, the NMA for anxiety included 47 study entries (n = 3,311) covering 9 interventions, comprising 27 pairwise comparisons, of which 18 were direct comparisons.

### 3.3 A network meta-analysis of efficacy in depression and anxiety

Across the included studies, various rating scales were utilized. Following data harmonization, higher scores consistently indicated greater symptom severity; therefore, a lower (negative) effect size reflects greater improvement.

**Figure 4:**
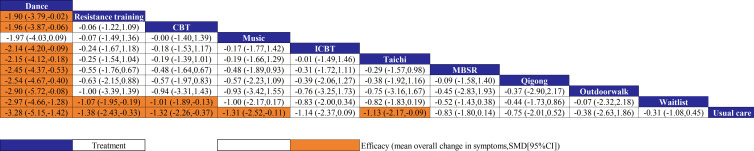
Network league table of pairwise comparisons for depressive symptoms.

Pairwise comparisons for depression were as follows: As shown in the network league table (**Figure 4**), dance significantly reduced depressive symptoms compared with resistance training (SMD = −1.90, 95% CI: −3.79 to −0.02), CBT (SMD = −1.96, 95% CI: −3.87 to −0.06), ICBT (SMD = −2.14, 95% CI: −4.20 to −0.09), Tai Chi (SMD = −2.15, 95% CI: −4.12 to −0.18), MBSR (SMD = −2.45, 95% CI: −4.37 to −0.53), qigong (SMD = −2.54, 95% CI: −4.67 to −0.40), outdoor walking (SMD = −2.90, 95% CI: −5.72 to −0.08), waitlist (SMD = −2.97, 95% CI: −4.66 to −1.28), and usual care (SMD = −3.28, 95% CI: −5.15 to −1.42). However, no statistically significant difference was observed between dance and music (SMD = −1.97, 95% CI: −4.03 to 0.09). Resistance training and CBT both demonstrated significant advantages over waitlist (SMD = −1.07 and −1.01, respectively) and usual care (SMD = −1.38 and −1.32, respectively). Additionally, music and Tai Chi were significantly more effective than usual care (SMD = −1.31 and −1.13, respectively). No other comparisons yielded statistically significant differences.

**Figure 5:**
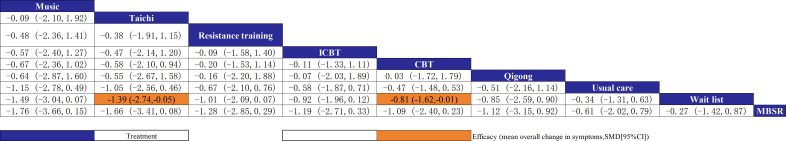
Network league table of pairwise comparisons for anxiety symptoms.

Pairwise comparisons for anxiety outcomes yielded the following results (**Figure 5**). Most comparisons did not reach statistical significance; only Tai Chi (SMD = −1.39, 95% CI: −2.74 to −0.05) and CBT (SMD = −0.81, 95% CI: −1.62 to −0.01) demonstrated statistically significant advantages over the waitlist control. Other highly ranked interventions, including music, resistance training, and ICBT, did not demonstrate statistically significant superiority over waitlist, usual care, or other active interventions.

### 3.4 Ranking of efficacy

The Surface Under the Cumulative Ranking (SUCRA) values and mean rankings under the random-effects model are summarized in **Tables 2**, **3**.

**Table 2:** SUCRA ranking for depression.

Treatment	SUCRA	Mean rank	No
Dance	98.5	1.2	1
Resistance training	67.5	4.3	2
CBT	65.6	4.4	3
Music	64.2	4.6	4
ICBT	56.7	5.3	5
Taichi	56.5	5.3	6
MBSR	43.0	6.7	7
Qigong	40.4	7	8
Outdoor walk	31.0	7.9	9
Wait list	18.5	9.2	10
Usual care	8.3	10.2	11

CBT, Cognitive Behavioral Therapy; MBSR, Mindfulness-Based Stress Reduction; ICBT, Internet-delivered Cognitive Behavioral Therapy.

**Table 3:** SUCRA ranking for anxiety.

Treatment	SUCRA	Mean rank	No
Music	78.9	2.7	1
Taichi	77.1	2.8	2
Resistance training	62.8	4	3
ICBT	59.7	4.2	4
CBT	56.6	4.5	5
Qigong	56.3	4.5	6
Usual care	31.8	6.5	7
Wait list	15.3	7.8	8
MBSR	11.4	8.1	9

CBT, Cognitive Behavioral Therapy; MBSR, Mindfulness-Based Stress Reduction; ICBT, Internet-delivered Cognitive Behavioral Therapy.

For depression, Dance exhibited the highest SUCRA (98.5%), ranking first among all interventions, followed by Resistance training (67.5%), CBT (65.6%), and Music (64.2%). ICBT (56.7%) and Taichi (56.5%) demonstrated moderate efficacy, while MBSR (43.0%), Qigong (40.4%), and Outdoor walk (31.0%) ranked lower. Waitlist control (18.5%) and Usual care (8.3%) demonstrated the lowest effectiveness.

For anxiety, Music exhibited the highest SUCRA (78.9%), ranking first among all interventions, closely followed by Taichi (77.1%). Resistance training (62.8%), ICBT (59.7%), CBT (56.6%), and Qigong (56.3%) demonstrated moderate efficacy. Usual care (31.8%) and Waitlist control (15.3%) ranked lower, while MBSR showed the least effectiveness with a SUCRA of 11.4% and ranked last.

Overall, while SUCRA scores provide a useful probability hierarchy, the network league tables emphasize that absolute rankings must be interpreted with clinical caution due to the overlapping confidence intervals among the top modalities.

### 3.5 Publication bias

Global inconsistency tests were non-significant for both networks, indicating that direct and indirect evidence were broadly consistent at the network level (depression: χ² = 18.01, p = 0.323; anxiety: χ² = 6.76, p = 0.873). This supports the overall credibility of the NMA estimates.

Node-splitting analysis, however, identified localized inconsistencies in both networks. In the depression network, three comparisons showed statistically significant divergence between direct and indirect estimates: waitlist versus resistance training (p < 0.001), CBT versus ICBT (p = 0.016), and usual care versus resistance training (p = 0.031). The resistance training inconsistency likely reflects heterogeneity in training protocols and intensity across contributing trials. In the anxiety network, four comparisons showed significant local inconsistency: CBT versus ICBT (p < 0.001), usual care versus Tai Chi (p = 0.002), usual care versus Music (p = 0.019), and waitlist versus Qigong (p = 0.048). These findings suggest that transitivity assumptions may not fully hold for these specific comparisons, and network estimates involving these pairs should be interpreted with additional caution. Affected comparisons were downgraded for inconsistency in the GRADE certainty assessment.

For the depression network, the comparison-adjusted Egger regression test, applied across all pairwise contrasts (k = 76), yielded a statistically significant intercept (intercept = 2.40, SE = 1.08, t_74_ = 2.23, p = 0.029), confirming the presence of small-study effects. Comparisons evaluated predominantly in small trials—particularly Dance (k = 4, all arms n < 40) and Music (k = 7, predominantly small)—are most plausibly implicated. For the anxiety network, the same test (k = 43 contrasts) yielded a non-significant result (intercept = 1.00, SE = 1.61, t_41_= 0.62, p = 0.538), providing no evidence of small-study effects. The top-ranked anxiety interventions, Music and Tai Chi, are therefore less susceptible to inflation from publication bias, lending comparatively greater credibility to their SUCRA rankings than to the depression rankings.

**Figure 6:**
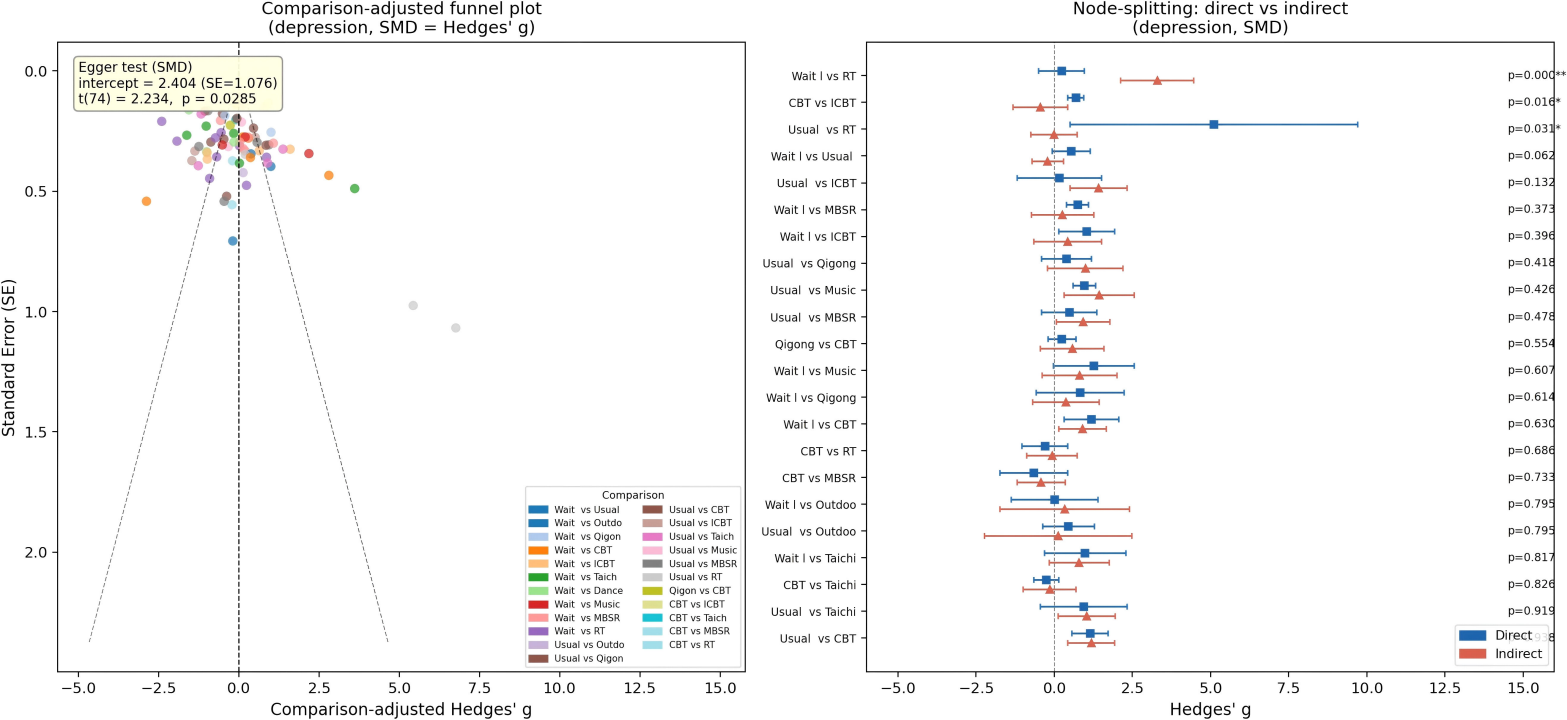
Comparison-adjusted funnel plot and node-splitting analysis for the depression network.

**Figure 7:**
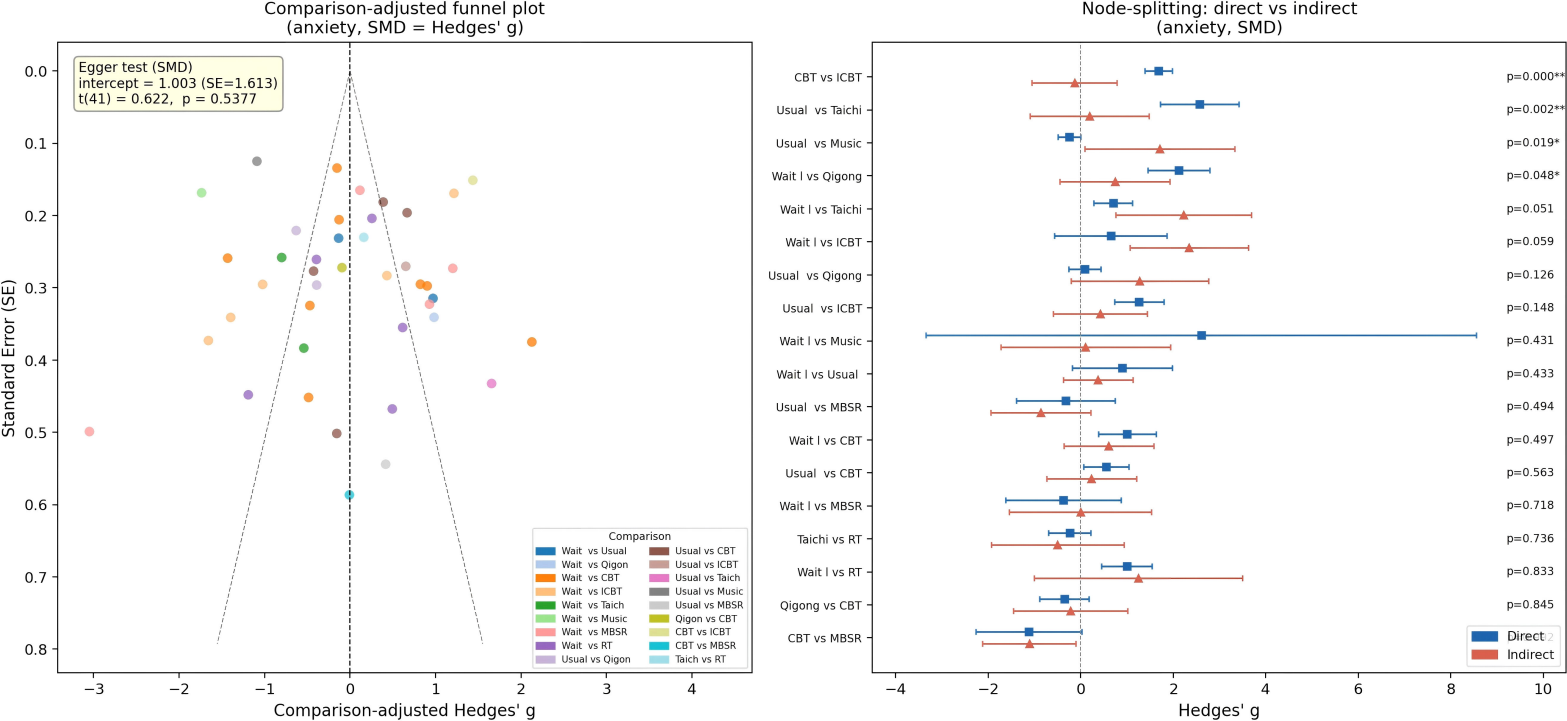
Comparison-adjusted funnel plot and node-splitting analysis for the anxiety network.

No trim-and-fill adjustment was performed for either network, as this method assumes a single symmetric funnel and lacks a validated extension to multi-treatment network geometry (19, 20). The significant depression Egger result is reported as an unresolved limitation that should be considered when interpreting SUCRA rankings for Dance and Music in the depression outcome (**Figures 6**, **7**).

### 3.6 Certainty of evidence

The certainty of evidence for each primary comparison was assessed using the GRADE approach; results are summarized in **Table 4**.

**Table 4:** Certainty of evidence for primary comparisons — GRADE approach.

Comparison	K	Risk of bias	Inconsistency	Indirectness	Imprecision	Publication bias	SMD (95% CI)	Certainty of evidence
Outcome: Depressive symptoms | Comparator: usual care								
Dance vs usual care	4	Serious ↓^a^	—	Serious ↓^b^	Serious ↓^c^	Suspected ↓^d^	−3.28 (−5.15 to −1.42) ✓	Very low
Resistance training vs usual care	17	Serious ↓^e^	Serious ↓^p^	—	—	Not detected^f^	−1.38 (−2.43 to −0.33) ✓	Low
CBT vs usual care	15	Serious ↓^g^	—	—	—	Not detected^f^	−1.32 (−2.26 to −0.37) ✓	Moderate
Music vs usual care	7	Very serious ↓↓^h^	—	—	Serious ↓^i^	Suspected ↓^f2^	−1.31 (−2.52 to −0.11) ✓	Very low
Tai Chi vs usual care	9	Serious ↓^j^	—	—	Serious ↓^k^	Not detected^f^	−1.13 (−2.17 to −0.09) ✓	Low
ICBT vs usual care	7	Serious ↓^l^	Serious ↓^q^	—	Serious ↓^m^	Not detected^f^	−1.14 (−2.37 to 0.09) ✗	Very low
Outcome: Anxiety symptoms | Comparator: waitlist control °								
Music vs waitlist	6	Very serious ↓↓^h^	—	Serious ↓^b^	Very serious ↓↓^n^	Not detected^f3^	−1.49 (−3.04 to 0.07) ✗	Very low
Tai Chi vs waitlist	9	Serious ↓^j^	Serious ↓^r^	—	Serious ↓^k^	Not detected^f3^	−1.39 (−2.74 to −0.05) ✓	Very low
CBT vs waitlist	15	Serious ↓^g^	—	—	Serious ↓^k^	Not detected^f3^	−0.81 (−1.62 to −0.01) ✓	Low
Resistance training vs waitlist	17	Serious ↓^e^	—	—	Serious ↓^m^	Not detected^f3^	−1.01 (−2.09 to 0.07) ✗	Low
ICBT vs waitlist	7	Serious ↓^l^	Serious ↓^s^	—	Serious ↓^m^	Not detected^f3^	−0.92 (−1.96 to 0.12) ✗	Very low

✓statistically significant (95% CI excludes zero); ✗ not statistically significant (95% CI crosses zero). — = not downgraded. CBT, cognitive behavioral therapy; ICBT, internet-based CBT; SMD, standardized mean difference; CI, confidence interval; k, number of studies contributing to the intervention group. Global NMA inconsistency test: depression network non-significant; anxiety network global test non-significant. Local inconsistencies identified via node-splitting (see footnotes [p]–[s]). Comparisons not footnoted were not downgraded for inconsistency.

^a^ Dance — Serious RoB: 2/4 (50%) high risk for allocation concealment; blinding of participants/personnel rated unclear in all 4 studies (inherent limitation of physical interventions). Downgraded 1 level.

^b^ Serious indirectness: The NMA transitivity assumption requires that participants in Dance trials be clinically exchangeable with those in other intervention trials. However, systematic baseline differences are plausible — dance participants tend to be more physically mobile and socially engaged than those enrolled in ICBT or MBSR trials. This constitutes a transitivity concern and represents a valid source of indirectness in NMA-GRADE (consistent with BMJ 2024 NMA framework). Additionally, the mixed population (healthy older adults and those with mild symptoms) without stratified analysis adds further indirectness. Downgraded 1 level.

^c^ Dance — Serious imprecision: k = 4 direct studies; optimal information size not met; CI width = 3.73 SMD units. Downgraded 1 level.

^d^ Dance — Publication bias suspected: the comparison-adjusted Egger regression test for the depression network (k = 76 contrasts) detected significant small-study effects (intercept = 2.40, SE = 1.08, t_74_ = 2.23, p = 0.029). All four direct Dance trials were small (n < 40 per arm), making Dance the comparison most plausibly driving this asymmetry. Downgraded 1 level.

^e^ Resistance training — Serious RoB: 8/17 (47%) high risk for allocation concealment; 11/17 (65%) high risk for blinding of participants/personnel (treated as structural design limitation in exercise trials, not an additional downgrade beyond allocation). Downgraded 1 level.

^f^ Publication bias not detected: although the depression network Egger test was significant (p = 0.029), these comparisons are supported by larger and more methodologically diverse direct evidence bases (k = 9–17 for resistance training; k = 15 for CBT), making small-study bias less plausible as a primary driver. Not downgraded.

^f2^ Music (depression) — Publication bias suspected: consistent with the significant depression network Egger test (p = 0.029), Music trials were predominantly small (median n < 40 per arm, k = 7). No further downgrade applied as certainty is already Very low.

^f3^ Publication bias not detected: the comparison-adjusted Egger regression test for the anxiety network (k = 43 contrasts) yielded a non-significant result (intercept = 1.00, SE = 1.61, t_41_= 0.62, p = 0.538), indicating no evidence of small-study effects in this network. Not downgraded.

^g^ CBT — Serious RoB: 6/15 (40%) high risk for allocation concealment; 3/15 (20%) high risk for blinding of participants; 4/15 (27%) high risk for blinding of outcome assessors. Downgraded 1 level.

^h^ Music — Very serious RoB: 5/7 (71%) high risk for allocation concealment AND 4/7 (57%) high risk for blinding of participants/personnel — a majority of contributing studies show critical flaws in two independent RoB domains simultaneously. Downgraded 2 levels.

^i^ Music depression — Serious imprecision: CI lower bound barely significant (−0.11); CI width = 2.41 SMD units; k = 7. Downgraded 1 level.

^j^ Tai Chi — Serious RoB: 5/9 (56%) high risk for allocation concealment. Blinding domains rated unclear (not high) across all 9 studies, reflecting the inherent difficulty of blinding mind-body interventions rather than confirmed bias. Downgraded 1 level for allocation concealment only.

^k^ Tai Chi — Serious imprecision: CI lower bound close to null (depression: −0.09; anxiety vs waitlist: −0.05); CI width is wide relative to effect magnitude. Downgraded 1 level.

^l^ ICBT — Serious RoB: 2/7 (33%) high risk for allocation concealment; 2/7 (33%) high risk for blinding of participants. Downgraded 1 level.

^m^ Serious imprecision: 95% CI crosses zero; effect estimate does not reach statistical significance under the random-effects model. Downgraded 1 level.

^n^ Music anxiety — Very serious imprecision: 95% CI crosses zero (−3.04 to 0.07); CI width = 3.11 SMD units; effect is not statistically significant; k = 6 direct studies with limited direct anxiety comparisons. Downgraded 2 levels. Note: Music SUCRA = 78.9% (rank 1) is driven predominantly by indirect NMA evidence and should not be interpreted as demonstrated superiority.

^o^ Comparator note (anxiety): When compared against usual care (rather than waitlist), Tai Chi (SMD = −1.05, 95% CI: −2.56 to 0.46) and CBT (SMD = −0.47, 95% CI: −1.48 to 0.53) do not reach statistical significance. Statistically significant advantages are only demonstrated against waitlist control. Clinicians should interpret anxiety efficacy estimates with reference to this comparator distinction.

^p^ Resistance training — Serious inconsistency: node-splitting analysis identified statistically significant local inconsistency in both waitlist versus RT (p < 0.001) and usual care versus RT (p = 0.031), suggesting that direct and indirect evidence for RT do not converge. This likely reflects heterogeneity in training intensity, protocol duration, and comparator definitions across contributing trials. Downgraded 1 level.

^q^ ICBT (depression) — Serious inconsistency: CBT versus ICBT showed significant local inconsistency (p = 0.016). As the network estimate for ICBT versus usual care relies substantially on indirect evidence routed through CBT, this inconsistency compromises the reliability of that estimate. Downgraded 1 level.

^r^ Tai Chi (anxiety) — Serious inconsistency: node-splitting identified significant local inconsistency in usual care versus Tai Chi (p = 0.002), directly affecting the Tai Chi network estimate. Downgraded 1 level.

^s^ ICBT (anxiety) — Serious inconsistency: CBT versus ICBT showed highly significant local inconsistency (p < 0.001). As the anxiety network estimate for ICBT versus waitlist routes substantially through CBT as an intermediary, this inconsistency undermines estimate reliability. Downgraded 1 level.

For depressive symptoms (comparator: usual care), resistance training (SMD = −1.38, 95% CI: −2.43 to −0.33) and CBT (SMD = −1.32, 95% CI: −2.26 to −0.37) were the highest-certainty comparisons. CBT was rated moderate certainty, downgraded once for serious risk of bias. Resistance training was rated low certainty, downgraded for serious risk of bias and serious local inconsistency (waitlist and usual care vs RT both p ≤ 0.031). Dance (SMD = −3.28, 95% CI: −5.15 to −1.42) was rated very low certainty, downgraded for serious risk of bias, serious indirectness (transitivity concerns from systematic baseline differences across trial populations), serious imprecision (k = 4; CI width = 3.73 SMD units), and suspected publication bias (Egger p = 0.029, all contributing trials small). Music (SMD = −1.31, 95% CI: −2.52 to −0.11) was similarly rated very low certainty, downgraded for very serious risk of bias (5/7 studies high-risk allocation concealment; 4/7 high-risk participant blinding), serious imprecision, and suspected publication bias. Tai Chi (SMD = −1.13, 95% CI: −2.17 to −0.09) was rated low certainty, downgraded for serious risk of bias and serious imprecision. ICBT (SMD = −1.14, 95% CI: −2.37 to 0.09) was rated very low certainty, downgraded for serious risk of bias, serious imprecision, and serious inconsistency (CBT vs ICBT p = 0.016); notably, ICBT did not reach statistical significance.

For anxiety symptoms (comparator: waitlist control), all comparisons were rated low or very low certainty. Tai Chi (SMD = −1.39, 95% CI: −2.74 to −0.05) and CBT (SMD = −0.81, 95% CI: −1.62 to −0.01) were the only interventions achieving statistical significance against waitlist control; both were rated low certainty, downgraded for serious risk of bias and serious imprecision. Tai Chi was additionally downgraded to very low certainty for serious local inconsistency (usual care vs Tai Chi, p = 0.002). Music (SMD = −1.49, 95% CI: −3.04 to 0.07) was rated very low certainty, downgraded for very serious risk of bias, very serious imprecision, and serious inconsistency (usual care vs Music, p = 0.019); its effect did not reach statistical significance. Resistance training (SMD = −1.01, 95% CI: −2.09 to 0.07) and ICBT (SMD = −0.92, 95% CI: −1.96 to 0.12) were rated very low certainty, downgraded for serious risk of bias, serious imprecision, and—for ICBT—serious inconsistency (CBT vs ICBT, p < 0.001); neither reached statistical significance. Importantly, when compared against usual care rather than waitlist control, neither Tai Chi nor CBT retained statistical significance, a distinction clinicians should consider when interpreting these findings.

Across both outcomes, inconsistency was the primary new downgrading domain identified in this analysis, affecting resistance training and ICBT for depression, and Tai Chi and ICBT for anxiety, based on localized node-splitting results (see Section 3.5). Publication bias was an additional concern for Dance and Music in the depression network, given the significant network-level Egger test (p = 0.029) and the exclusively small-trial evidence bases for these interventions. These two factors—inconsistency and small-study effects—constitute the main threats to the validity of the network estimates for the top-ranked interventions, and should inform both clinical application and future research priorities.

## 4 Discussion

To the best of our knowledge, this study represents the most comprehensive network meta-analysis (NMA) conducted to date that systematically evaluates the comparative effectiveness of diverse non-pharmacological interventions, including structured physical exercises, mind-body practices, psychological therapies, and arts-based modalities, in alleviating depression and anxiety among older adults. Late-life depression and anxiety represent profound public health challenges that are often exacerbated by medical comorbidities, functional decline, and pervasive social isolation. (104, 105). Given that older adults are highly susceptible to the adverse effects of psychotropic medications, including the risk of falls, polypharmacy interactions, and cognitive blunting associated with prolonged use of antidepressants and benzodiazepines, prioritizing robust non-pharmacological interventions is of paramount clinical importance (106, 107). By applying a rigorous random-effects model to account for the substantial clinical and methodological heterogeneity across 83 RCTs, our findings provide a highly nuanced perspective. Our updated analysis reveals that multimodal physical interventions (specifically dance) and mind-body/arts-based therapies (music and Tai Chi) demonstrate the highest probability of efficacy for depression and anxiety, respectively. However, the wide and overlapping confidence intervals observed in our network league tables emphasize that no single intervention possesses absolute universal superiority, underscoring the critical need for personalized, patient-centered care strategies.

Under the random-effects framework, dance ranked highest among the interventions for alleviating depression (SUCRA = 98.5%) and demonstrated statistically significant superiority over several established modalities, including resistance training and cognitive behavioral therapy (CBT). The marked efficacy of dance may be attributed to its unique, multifaceted nature. Unlike singular exercise forms, dance functions as a complex “multi-task” intervention that simultaneously integrates aerobic physical exertion, continuous cognitive stimulation (e.g., memorizing steps, rhythm adaptation, and spatial coordination), and rich social interaction. (108, 109). This biopsychosocial complexity makes it uniquely suited for older adults, who frequently suffer from concurrent executive dysfunction, which is a core feature of the vascular depression hypothesis (110). Neurologically, multimodal interventions like dance have been shown to robustly upregulate neurotrophic factors, particularly brain-derived neurotrophic factor (BDNF), thereby facilitating hippocampal neurogenesis, enhancing structural neuroplasticity, and significantly improving both mood and cognitive function (111–113). Furthermore, the communal aspect of dance directly combats social isolation, a primary psychosocial driver of late-life psychological distress (114). While dance demonstrated less pronounced effects in anxiety outcomes, its marked success in reducing depressive symptoms highlights the therapeutic superiority of combining motor, cognitive, and social elements into a single cohesive activity. It should be noted, however, that the certainty of Dance’s depression estimate is rated very low, primarily due to the small direct evidence base (k = 4), transitivity concerns, and significant small-study effects detected in the depression network (Egger test p = 0.029). Clinicians should therefore interpret Dance’s top ranking as an indication of promise rather than established superiority.

Similarly, resistance training has demonstrated substantial benefits, maintaining a strong comparative standing for both depression (SUCRA = 67.5%) and anxiety (SUCRA = 62.8%). The psychiatric benefits of resistance training in geriatrics extend well beyond traditional monoamine regulation (e.g., serotonin and dopamine synthesis) (115). Aging is intrinsically linked to sarcopenia and physical frailty, conditions that share a bidirectional pathogenic relationship with depression and anxiety (116). Resistance training directly disrupts this detrimental cycle. Physiologically, skeletal muscle contraction during progressive resistance training stimulates the release of myokines (such as irisin and insulin-like growth factor-1) and attenuates systemic low-grade inflammation, both of which represent neuroprotective mechanisms that are heavily implicated in mood regulation (117, 118). Psychologically, resistance training rapidly enhances physical self-efficacy, restores functional independence, and rebuilds self-esteem—factors that are frequently diminished in frail older adults (119).

For anxiety management, music therapy (SUCRA = 78.9%) and Tai Chi (SUCRA = 77.1%) ranked as the most promising interventions. The robust anxiolytic effects of these modalities likely stem from their direct modulation of the autonomic nervous system and deep brain networks. Music therapy, whether through passive listening or active group engagement, effectively targets the limbic system and has been documented to modulate the Default Mode Network (DMN), thereby reducing maladaptive cognitive rumination commonly seen in anxious patients (120). Additionally, music down-regulates the hypothalamic-pituitary-adrenal (HPA) axis, significantly reducing circulating cortisol levels and mitigating physiological markers of distress (121, 122). Similarly, Tai Chi functions as a form of moving meditation. By coupling slow, controlled biomechanical movements with deep diaphragmatic breathing and focused attention, Tai Chi shifts the autonomic balance toward parasympathetic dominance, inducing a state of profound physiological relaxation (123). Notably, Tai Chi and CBT were the only interventions to demonstrate statistically significant advantages over the waitlist control for anxiety; however, the certainty of Tai Chi’s anxiety estimate was rated very low following downgrading for local inconsistency (usual care vs Tai Chi, p = 0.002), serious risk of bias, and serious imprecision. Its ranking therefore reflects probabilistic promise rather than confirmed efficacy. Given its deep cultural roots, high accessibility, and minimal biomechanical impact on aging joints, Tai Chi represents a highly sustainable anxiolytic strategy for older adults (124).

Despite the ascendance of physical and arts-based interventions in our random-effects rankings, traditional and digital psychological therapies—namely CBT and ICBT—continued to demonstrate strong and consistent comparative efficacy across both psychological domains. Older adults with clinical depression and anxiety often exhibit maladaptive cognitive patterns, such as catastrophic thinking and negative overgeneralization. CBT directly and systematically targets these irrational schemas, restructuring them into constructive cognitions to disrupt the emotional feedback loop (125). While ICBT occupied higher rankings under initial fixed-effects assumptions, the random-effects model shifted it to a moderate standing for both outcomes. Furthermore, significant local inconsistency between CBT and ICBT (depression: p = 0.016; anxiety: p < 0.001) indicates that direct and indirect evidence for ICBT do not converge reliably, resulting in very low certainty ratings for ICBT in both outcomes. ICBT’s practical value therefore rests less on its SUCRA rank and more on its scalability and accessibility advantages. Nonetheless, ICBT remains a critical therapeutic asset. It overcomes significant systemic barriers to conventional mental healthcare—such as geographic isolation, mobility constraints, perceived stigma, and the severe shortage of geriatric mental health professionals—providing a highly scalable and cost-effective alternative for the digitalizing aging population (126, 127). In contrast, unstructured interventions, such as outdoor walking, consistently ranked lower, suggesting that aging populations may require more structured, actively guided, or socially integrated frameworks to achieve significant mental health improvements.

### 4.1 Clinical implications

The findings of this random-effects network meta-analysis have significant clinical implications. Crucially, the wide and overlapping confidence intervals observed in our network league tables indicate that strict adherence to absolute SUCRA rankings is clinically inappropriate. Rather than seeking a definitive “best” treatment, clinicians should adopt a shared decision-making approach within a collaborative stepped-care model (128). The choice of non-pharmacological intervention must be highly individualized, aligning with the older adult’s physical capabilities, baseline cognitive function, personal preferences, and socioeconomic resources. For example, socially isolated older adults with preserved mobility may derive maximum benefit from group dance classes; individuals with physical frailty or severe osteoarthritis may respond best to seated music therapy or Tai Chi; and those who are housebound but digitally literate could be ideal candidates for ICBT. Integrating these evidence-based non-pharmacological options into routine primary care and community senior centers is essential for comprehensive geriatric mental health management. Crucially, the GRADE certainty ratings presented in **Table 4** indicate that only CBT achieves moderate certainty for depression, while resistance training was downgraded to low certainty following detection of local inconsistency in resistance training comparisons. Dance, ICBT (depression), Tai Chi (anxiety), and ICBT (anxiety) are all rated very low certainty. All remaining comparisons are rated low. Clinicians should weight treatment recommendations according to both SUCRA probability rankings and underlying evidence certainty, avoiding over-reliance on high SUCRA values derived from limited or methodologically compromised evidence bases.

### 4.2 Limitations

Several important limitations of this study must be acknowledged, providing critical context for interpreting our findings. First, due to inconsistent reporting of individual patient-level data across the primary trials, we were unable to strictly stratify populations by baseline symptom severity (e.g., healthy older adults versus those with clinically diagnosed mild-to-moderate symptoms). Pooling these diverse baseline populations may obscure critical clinical nuances regarding how specific interventions perform at different symptom thresholds. Second, while the SUCRA metric provides a statistically intuitive hierarchy of efficacy probability, the wide and overlapping confidence intervals identified in our random-effects league tables indicate significant clinical and methodological heterogeneity across the network. Consequently, declaring any single intervention as definitively “most effective” is both statistically and clinically unwarranted. Third, the comparison-adjusted Egger test detected significant small-study effects in the depression network (p = 0.029), indicating that smaller trials tend to report larger depression effect sizes. This is of particular concern for Dance and Music, whose direct evidence bases consist almost exclusively of small trials; because no validated trim-and-fill extension exists for network meta-analysis, the magnitude of potential inflation remains unquantified. Importantly, no corresponding small-study effect was detected in the anxiety network (p = 0.538), suggesting that SUCRA rankings for Music and Tai Chi in the anxiety outcome are less susceptible to this bias. In addition, localized inconsistencies detected via node-splitting in both networks—particularly for resistance training and ICBT comparisons in depression, and for Tai Chi, ICBT, and Music comparisons in anxiety—indicate that certain network estimates rely on indirect evidence paths that do not fully converge with direct evidence, further warranting cautious interpretation. Fourth, the included RCTs exhibited considerable heterogeneity in intervention protocols, including variations in session frequency, dosage, practitioner expertise, and duration, which likely contributed to the wide confidence intervals. Fifth, the GRADE certainty ratings indicate that the evidence base for several top-ranked interventions—particularly Dance and Music for depression, and all interventions for anxiety—is of low or very low certainty. This reflects the small number of direct comparative trials, high rates of allocation concealment failures, and the inherent impossibility of participant blinding in physical and arts-based interventions. These ratings do not invalidate the observed effects but signal that future well-designed RCTs could substantially change current estimates. Finally, methodological concerns, particularly inadequate reporting of allocation concealment and blinding of outcome assessors in a subset of trials, underscore the persistent need for higher-quality evidence. Based on these findings, we strongly recommend that future multi-arm RCTs adhere to rigorous methodological standards, prioritize direct head-to-head comparisons of prominent non-pharmacological modalities, and consistently report patient-level baseline data to facilitate robust subgroup analyses.

## 5 Conclusion

This NMA demonstrates that dance ranks highest for alleviating depressive symptoms in older adults (SUCRA = 98.5%), followed by resistance training and CBT. For anxiety, music and Tai Chi demonstrate the highest probability of efficacy (SUCRA = 78.9% and 77.1%, respectively), with Tai Chi and CBT being the only interventions to achieve statistically significant advantages over waitlist control. ICBT and CBT maintain consistent comparative standings across both outcomes and offer scalable, accessible delivery modes particularly suited for older adults with mobility limitations, although ICBT’s certainty ratings are very low owing to significant local inconsistency between direct and indirect evidence. MBSR and outdoor walking demonstrated limited comparative effectiveness. Given the substantial heterogeneity and low-to-very-low certainty of evidence for several top-ranked interventions, these findings should be interpreted as probabilistic guidance to inform individualized, stepped-care decision-making rather than definitive treatment hierarchies. Future high-quality RCTs with standardized protocols and direct head-to-head comparisons are needed to strengthen the evidence base.

### 5.1 Relevance for clinical practice

In summary, dance and resistance training should be prioritized for older adults with depression who have adequate mobility, given their high SUCRA rankings and established neurobiological mechanisms. For anxiety management, music therapy and Tai Chi represent the most promising options, with Tai Chi uniquely supported by statistical significance against waitlist control. ICBT and CBT remain strongly recommended across both outcomes owing to their scalability, accessibility, and demonstrated clinical utility—particularly for individuals with mobility constraints or in underserved settings, where CBT’s moderate certainty for depression provides the strongest evidence base. Clinicians should reconsider reliance on less structured approaches such as outdoor walking, which showed limited comparative effectiveness. All treatment decisions should integrate SUCRA rankings with GRADE certainty ratings and individual patient factors including physical capacity, cognitive function, and accessibility.

## Data Availability

The original contributions presented in the study are included in the article/supplementary material. Further inquiries can be directed to the corresponding author.
